# High Energy Channeling and Malleable Transition States: Molecular Dynamics Simulations and Free Energy Landscapes for the Thermal Unfolding of Protein U1A and 13 Mutants

**DOI:** 10.3390/biom12070940

**Published:** 2022-07-04

**Authors:** Na Le Dang, Anne M. Baranger, David L. Beveridge

**Affiliations:** Department of Chemistry and Molecular Biophysics Program, Wesleyan University, Middletown, CT 06459, USA; ldang@wesleyan.edu (N.L.D.); abaranger@berkeley.edu (A.M.B.)

**Keywords:** Molecular dynamics, protein unfolding, transition state, U1A

## Abstract

The spliceosome protein U1A is a prototype case of the RNA recognition motif (RRM) ubiquitous in biological systems. The in vitro kinetics of the chemical denaturation of U1A indicate that the unfolding of U1A is a two-state process but takes place via high energy channeling and a malleable transition state, an interesting variation of typical two-state behavior. Molecular dynamics (MD) simulations have been applied extensively to the study of two-state unfolding and folding of proteins and provide an opportunity to obtain a theoretical account of the experimental results and a molecular model for the transition state ensemble. We describe herein all-atom MD studies including explicit solvent of up to 100 ns on the thermal unfolding (UF) of U1A and 13 mutants. Multiple MD UF trajectories are carried out to ensure accuracy and reproducibility. A vector representation of the MD unfolding process in RMSD space is obtained and used to calculate a free energy landscape for U1A unfolding. A corresponding MD simulation and free energy landscape for the protein CI2, well known to follow a simple two state folding/unfolding model, is provided as a control. The results indicate that the unfolding pathway on the MD calculated free energy landscape of U1A shows a markedly extended transition state compared with that of CI2. The MD results support the interpretation of the observed chevron plots for U1A in terms of a high energy, channel-like transition state. Analysis of the MDUF structures shows that the transition state ensemble involves microstates with most of the RRM secondary structure intact but expanded by ~14% with respect to the radius of gyration. Comparison with results on a prototype system indicates that the transition state involves an ensemble of molten globule structures and extends over the region of ~1–35 ns in the trajectories. Additional MDUF simulations were carried out for 13 U1A mutants, and the calculated φ-values show close accord with observed results and serve to validate our methodology.

## 1. Introduction

Protein folding and unfolding at the molecular level has been elucidated to a considerable extent by studies on simple two-state processes, which typically have relatively narrow and sharply peaked activation barriers between native (N) and denatured (D) states on the free energy landscape [1] An interesting variation on the theme of two-state protein folding has been observed for the folding/unfolding of the spliceosome protein U1A, which shows that the folding/unfolding of U1A is essentially two-state but takes place by “high energy channeling” via a broad and flat activation barrier and a “malleable” transition state [2]. Further understanding of these phenomena at the molecular level is an important next step.

The synergy between molecular simulations and experiments has been evolving rapidly with the improvement of force fields and access to high-performance computing. Refs. [2,3,4,5,6] Molecular dynamics (MD) simulations have recently proved to be quite successful in obtaining computational models for two-state protein folding [7,8] and unfolding [9,10]. Daggett and coworkers have published extensively on thermal unfolding (UF) of small proteins using MD [11], henceforth abbreviated MDUF, and elucidated transition state processes from a representation of MDUF trajectories in root-mean-square-deviation (RMSD) space [12,13]. This methodology provides an opportunity to investigate the extent to which MD simulations can account theoretically for the particular behavior observed for U1A and investigate the nature of the transition state ensemble at the molecular level. In the course of this study, we have combined previous procedures into a practical method for calculating a statistical free energy landscape in RMSD space from MD simulations.

We describe, herein, all-atom MDUF simulations including solvent on U1A and 13 mutants. Our simulations address the following specific questions: (a) Does the MDUF energy landscape for U1A support the interpretation of kinetic experiments in terms of a high energy channel and malleable transition state? (b) What is the molecular model obtained from MDUF for the transition state ensemble? (c) What is the mechanism of unfolding indicated by the MDUF simulations and is this relevant to folding pathways? (d) What elements of secondary structure constitute the nucleation site, and what residues are involved in the folding nucleus? (e) How do the MD results on U1A unfolding compare with mechanisms and structures derived in previous experimental and theoretical studies on U1A? This project builds on our previous studies of all-atom, explicit solvent MD on the equilibrium dynamics of U1A and F56 mutants [14,15,16,17,18].

## 2. Background

**Figure 1:**
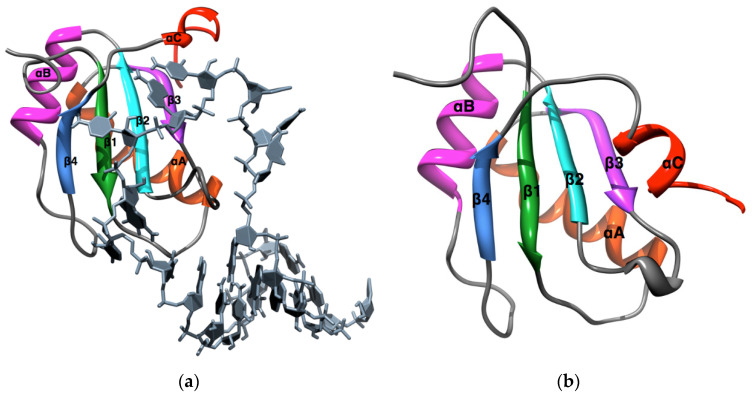
(**a**) Crystal Structure of the protein U1A-Stem loop 2 RNA Complex [24], with secondary structure and other elements annotated; (**b**) NMR solution structure of uncomplexed U1A [23], structure #5 from the ensemble of pdb #1fht.

The protein U1A belongs to a family of RNA recognition motifs (RRM), one of the most abundant protein domains in eukaryotes [19]. Proteins containing RRMs participate in all steps of gene expression and RNA processing and bind to single-stranded RNAs of diverse sequences in a variety of structural contexts [20,21]. The crystal structure of U1A complexed with a 21-mer stem-loop RNA [22] shows a four stranded, antiparallel β-sheet flanked by two α-helices, characteristic of an RRM, and organized according to the pattern β_1_α_A_β_2_β_3_α_B_β_4_(α_C_) with respect to sequence and secondary structure (Figure 1a). In the native tertiary structure, the RRM is organized as an antiparallel β-sheet β_4_β_1_β_3_β_2_ with α_A_ and α_B_ as supports. The structure of uncomplexed U1A in solution [23] (Figure 1b), is similar to that of the complex with RNA with the exception of the C-terminal helix α_C_, which folds over and obstructs the access of ligand RNA to the RRM. The intrinsic stability of the RRM in U1A and the dynamics of α_C_ and also loop 3 contribute significantly to U1A-RNA recognition and are thus of particular interest.

Silow and Oliveberg [25] measured experimentally the in vitro kinetics of U1A folding and unfolding as a function of denaturant concentration using stopped flow and analyzed the results with chevron plots [1]; linearity in the arms of the chevron is diagnostic of a two-state process [26]. In the case of U1A, a deviation from simple two-state behavior was indicated by a slight but significant curvature in the arms. This was interpreted based on β-values [1] as evidence for large movements and changes in solvent exposure in the transition state structures along the top of a very broad free energy barrier. Further experimental studies of the formation and growth of the folding nucleus of U1A based on φ-value analysis were reported by Ternstrom et al. [27] and supported the delocalized nature of the transition state. A model was proposed, in which the folding nucleus involved β_2_, β_3_ and α_A_ of the RRM. A previous theoretical study of wild-type U1A and various mutants at a coarse-grained level has been reported based on a variational free energy functional theory [28] and provided a description of the growth of the folding nucleus in terms of residue-based isodensity surfaces [29]. Lindberg and Oliveberg [30] discussed the folding of U1A and other proteins in terms of modular assemblies of competing two-strand-helix nucleation motifs and pointed out that pathway malleability may be a more general phenomenon in protein folding and induced in a variety of ways. This is supported by recent work on the homeodomain superfamily [31].

Early studies of MD applied to protein unfolding were due to Daggett and Levitt [32,33], and the current state of the field has been recently reviewed [6,11,13,34]. Notably, an extensive database of protein dynamics has been created [35]. There have been a number of subsequent articles describing the use of MD to study the thermal unfolding of proteins and to characterize transition states [11]. Daggett and coworkers have defined a general protocol for MDUF simulations [36] and documented the applicability of MDUF to realistic protein unfolding, including the correspondence between thermal unfolding and chemical denaturation for small proteins [37], and that high temperature accelerates the unfolding of small proteins without essentially changing the pathway [38,39]. Results from 100 independent MDUF simulations on CI2 [40], showed that 5–10 MDUF trajectories are required to capture properly the average properties. Daggett and coworkers in a number of studies [11] have utilized the calculated pair-wise C^α^ RMSD matrix (2DRMSD) reduced by classical multidimensional scaling (MDS) [12,13,41], to represent a trajectory by plotting the vector components of the RMSD difference between successive MD structures (MD step vectors) in the RMSD space. In this method, the transitional region for protein unfolding is defined as the first point of exit from the native state ensemble, i.e., the first precipitous change in the RMSD between successive MDUF structures. This method is a type of leader algorithm [42] and has appeared elsewhere in the protein folding literature [43]. Stereoscopic views of MD step vector plots in the 3D RMSD space have been presented [13,44], but typically a 2D projection has been sufficient to elucidate the transition [12].

There is considerable literature on the determination of reaction coordinates for protein folding and unfolding. One line of approach involves using linear or non-linear MDS to reduce the MD trajectory to a representation in terms of order parameters (i.e., native contacts, the radius of gyration, etc.) in two or three dimensions that can be readily interpreted and used to calculate free energy landscapes and locate transition states [45,46,47,48]. A concern with this type of approach is that depending on the order parameter, MDS reduction may lead to an oversimplified free energy landscape, especially in the region of highly unfolded structures [49,50], but the specifics of this issue are unsettled [11] and await more comparative studies. More rigorous methods have been proposed [49,51], and enable analyses that are even finer-grained than the level of resolution of most experiments. Theoretically, structures that comprise the transition state ensemble should have an equal probability *P_fold_* to fold and unfold [52,53]. A critical consideration and further methodological developments based on *P_fold_* type approaches have been provided by Muff and Caflisch [43]. Several recent studies have reported transition state ensembles for proteins comprised of structures with *P_fold_* = 0.5 [13,54]. Beck and Daggett [13] demonstrated that the transition state ensemble obtained by the *RMSD* space method is quite similar to that of the computationally much more intensive *P_fold_* method, and showed that the transition state ensemble obtained by their method is quite similar to that based on structures which have an equivalent propensity to fold and unfold. Transition disconnectivity graphs [55] and complex network analysis [50] provide additional tools for detailed analysis of the energy and free energy landscapes, ofttimes used together with the above methods [49,51]. Daggett and coworkers have also proposed a comprehensive embedded 1D reaction coordinate for protein unfolding and folding [56].

## 3. Materials and Methods

### 3.1. Molecular Dynamics Simulations

The MD simulations in this study are carried out on an all atom, explicit solvent model of the system using the *AMBER* suite of programs [57]. Explicit consideration of solvent water and ions is necessary to describe the molecularity of water–protein hydrogen bonding and the hydrophobic effect [11]. The simulations are based on the protein force field *parm99SB* [58], which performed among the best in a recent study of the various options for MD on proteins [8]. The *TIP3P* force field [59] was used for water molecules and *ions08* [60] was used for monovalent ions. The system for simulation studies was configured under minimal neutralizing salt conditions, and comprised of protein, ~5000 water molecules, and eight Cl^−^ ions. All MDs were performed under constant volume (T, V, N) ensemble conditions with particle-mesh Ewald periodic boundary conditions. For the integration of the equations of motion, an MD time step of 1 fs was adopted. Our simulations follow closely the MDUF protocol of Beck and Daggett [36], with a water density of 0.829 gm/cc and a temperature of 498 K. All MD simulations were carried out from an energy minimized form of the NMR solution structure, of U1A, structure #5 of PDB ID #1fht (Figure 1b). The structures of the NMR ensemble are all very similar. To ensure accuracy and reproducibility, five independent thermal unfolding simulations with different initial velocity distributions were carried out.

### 3.2. Analysis

MD calculated free energy landscapes were obtained using a particular combination of two existing methodologies. The first method, as discussed above, is the representation of a simulation in terms of a plot of MD step vectors in RMSD space [12,13]. The native state is represented as a relatively tight cluster of step vectors. In a system with potentially extended activation barriers, the first point of exit from the native state ensemble defines the onset of the UF transition region, but a demarcation of the outer bound of the transition is also necessary (see below). The method is quite general and has been applied extensively to calculate free energy surfaces for proteins with respect to the first two eigenvectors of a principal component analysis (PCA) of a protein MD trajectory [61,62,63,64]. Here, a 2D grid is defined on a representation of a trajectory in PCA eigenvector space and the population of structures *P_ij_* associated with each grid element is determined. From this, normalized probabilities *P_ij_/P_0_* can be obtained, where *P_0_* is the maximum population. Statistical free energy is then calculated as *ΔF_ij_ = −RT ln (P_ij_/P_0_)* and displayed as a contour map. In PCA, one typically considers a subspace of the motions defined by the two most important principal components of the motions (the essential dynamics [61]) which typically accounts for >2/3 of the total motion and sometimes more, depending on the system. Similar applications of this approach have been described in terms of collective variables, such as native contacts [65,66] and radius of gyration R_g_ [13]. The free energy landscapes for this study are obtained in an analogous manner but are based on population maps of the terminals of MD step vectors in the 3D of RMSD space. An advantage of this approach is that 100% of the unfolding trajectory is represented, albeit in a space of reduced dimensionality.

## 4. Results and Discussion

**Figure 2:**
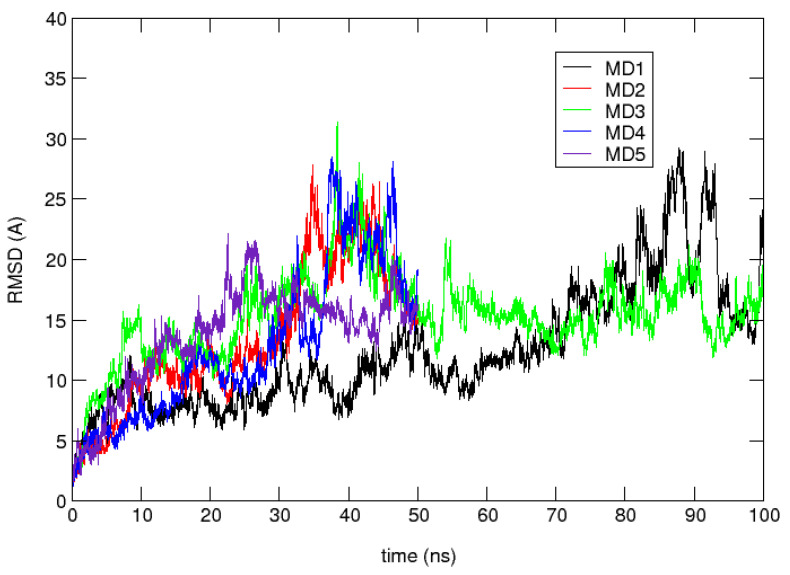
Root mean square deviation (RMSD) with respect to the initial structure as a function of time for 5 independent MDUF trajectories for U1A. Only MD3 and MD5 were extended; all were showing essentially similar behavior after 50 ns.

**Figure 3:**
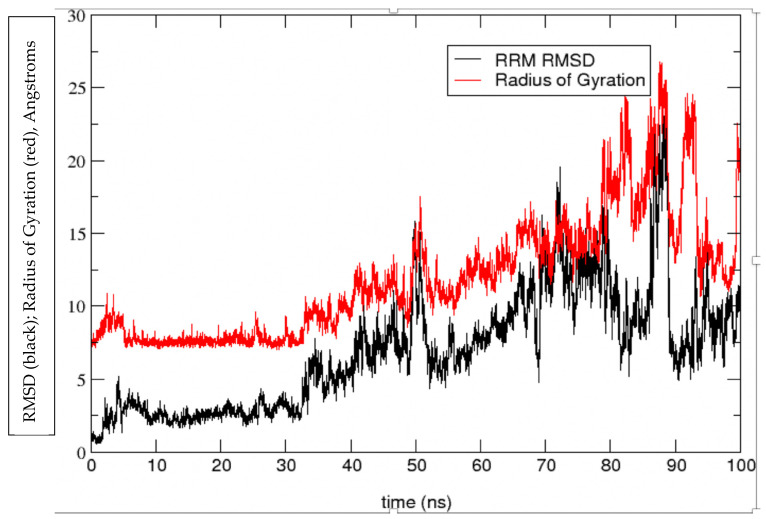
Radius of Gyration (red line) and RMSD (black line) of the RNA recognition motif of U1A with respect to the initial structure of MDUF simulation #1 on U1A.

Validation of the MDUF was first considered by plotting the C^α^ RMSD with respect to the initial MD structure and the corresponding radius of gyration R_g_ as a function of time. The results are shown in Figure 2 and Figure 3, respectively. The MD calculated C^α^ RMSD and R_g_ closely parallel one another, showing an initial rapid departure from the initial configuration, equilibration after ~10 ns to a meta-stable region of ~30 ns in which the MD structures cluster in the vicinity of ~7.5 Å rmsd. After ~30 ns, the protein unfolds more or less monotonically until 80 ns. From there on until the MD was terminated at 100 ns, the protein exhibits high amplitude oscillations in the range of 10 Å to 27 Å RMSD from the initial structure.

**Figure 4:**
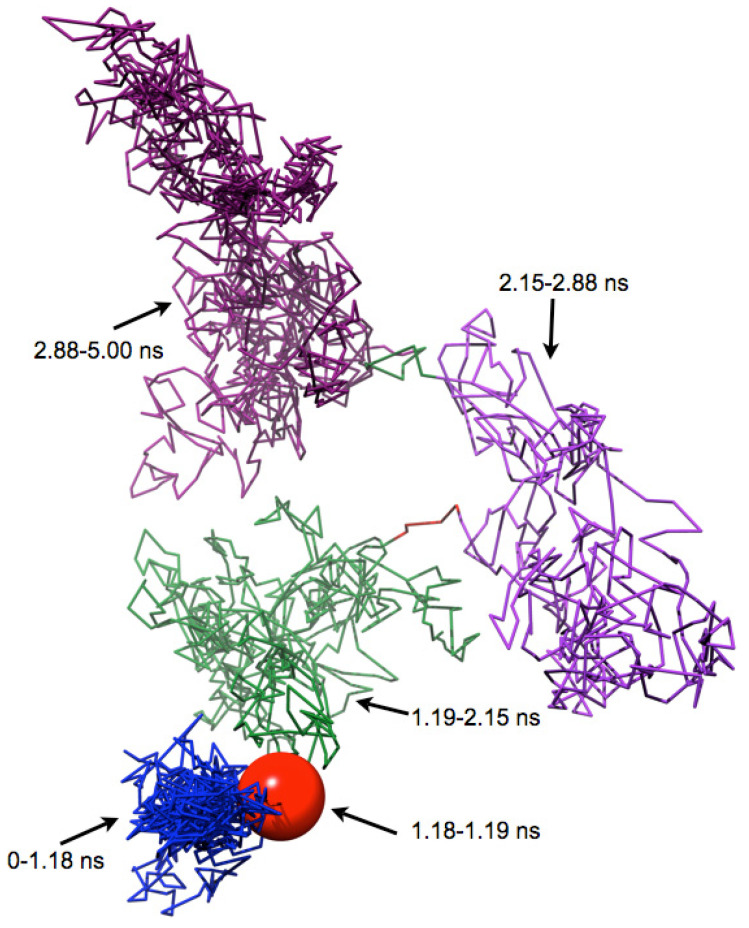
Vectorial representation of the first 5 ns of a 100 ns MDUF trajectory #1 in RMSD space obtained using the RMSD method of Daggett and coworkers [12,13]. The coloring distinguishes the various transitional intermediates.

**Figure 5:**
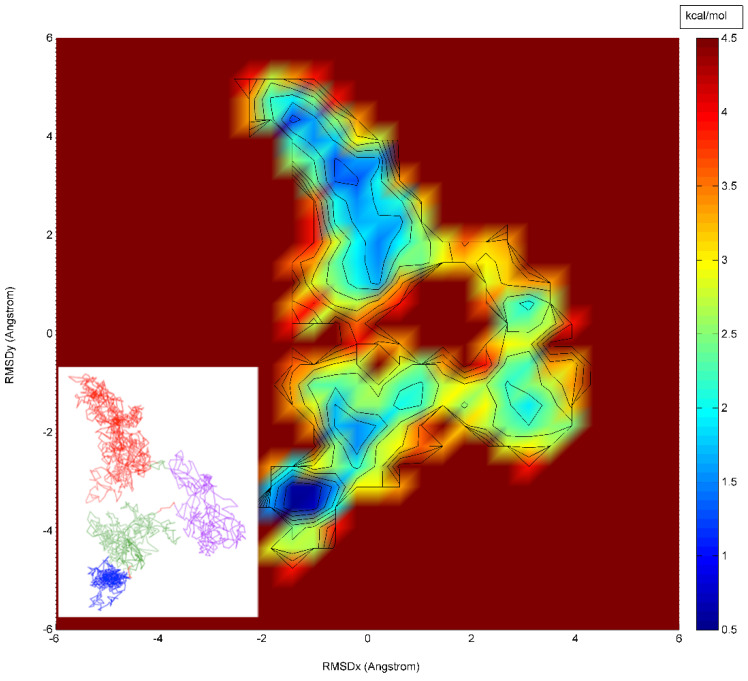
Calculated PCA free energy landscape for the first 5 ns of a 100 ns MDUF trajectory #1 on U1A. The (non-equilibrium) unfolding reaction coordinate based on the gradient of isoenergy contour lines is indicated as a dotted line. Color scheme: yellow to green to blue: high to medium to low free energies, respectively.

**Figure 6:**
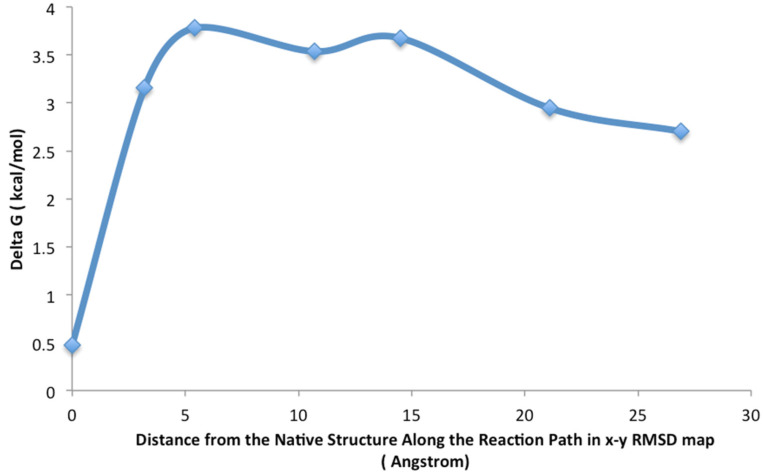
Calculated free energy profile for U1A unfolding with respect to the reaction coordinate indicated in Figure 4. The depth of the slight minimum after the activation barrier is ~0.2 kcal/mol and does not correspond to a thermally stable intermediate.

An RMSD vector representation of the MDUF trajectory for U1A in a 2D projection of the 3D RMSD space is shown in Figure 4. The onset of UF transition occurs in the region of ~1.18–1.19 ns (indicated as a red sphere). Using the methodology described in the preceding section, a population map was obtained and converted to a statistical free energy landscape. The result is presented as a contour map in Figure 5. This describes the MD UF transition as proceeding from the native state (lower left corner) over a free energy barrier and continuing along a relatively extended valley on the surface. A UF reaction coordinate is constructed from perpendiculars to the isoenergy contour lines. The free energy for the first 5 ns of MD simulated unfolding of U1A as a function of this coordinate is plotted in Figure 6. The plot indicates an activation free energy of 3.8 kcal/mol. Using the transition state theory for proteins [1] the *k_u_* is approximately 10^6^ exp(-ΔF/RT) which computes to 2.7 × 10^−3^ s^−1^. The free energy profile in Figure 6 for the first 5 ns of MDUF shows that the activation process is to be followed by a slight free energy minimum before unfolding further. However, the well depth of this minimum is only ~0.2 kcal/mol, so this does not correspond to a thermally stable intermediate. Thus, the MD UF results on U1A describe a two-state process.

**Figure 7:**
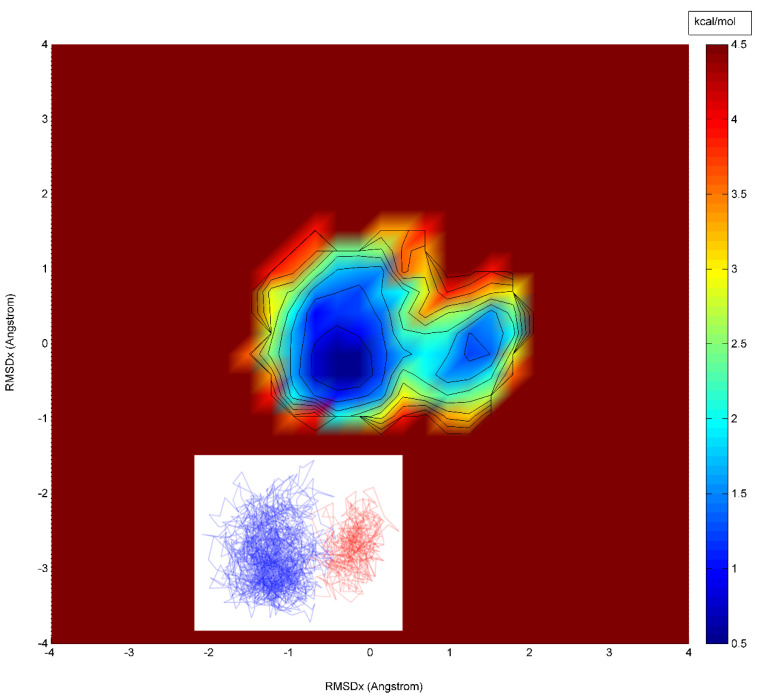
Calculated PCA free energy landscape for an MDUF trajectory on CI2. Color scheme: yellow to green to blue: high to medium to low free energies, respectively.

To confirm that our calculated unfolding pathway involves a high energy channel and extended transition state, we have taken as a control, the well-characterized [12] two-state protein CI2, and performed an analogous MDUF with a similar protocol to that used for U1A and calculated a corresponding free energy landscape (Figure 7). The result is clearly consistent with that of a simple two-state process, with a narrow and sharply peaked barrier to activation and the transition state covered in an interval of <1 ns. Comparing the free energy landscape calculated for U1A in Figure 5 with that of CI2 in Figure 7, the results indicate that the MDUF on U1A does indeed show a relatively extended transition state. Similar results were obtained from the analysis of our other four MDUF simulations on U1A.

**Figure 8:**
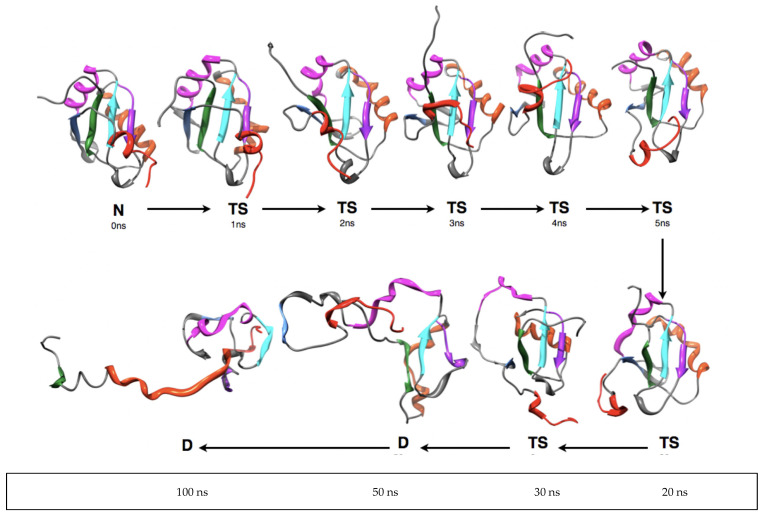
A series of MD snapshots from 100 ns of the MDUF trajectory #1 for U1A.

**Figure 9:**
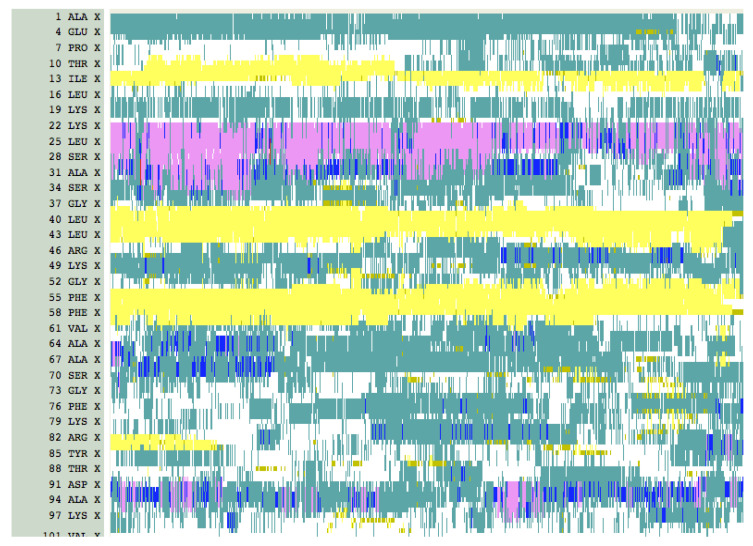
Evolution of secondary structure in the MDUF trajectory #1 of U1A. Key: Yellow: β-sheets. Red: α-helices. This plot was obtained using VMD [68].

A time series of structures from MDUF #1 on U1A is shown in Figure 8. Here, again similar results were obtained from our other four independent MDs on U1A. By 1 ns into the trajectory, helix α_C_ shows dynamical reorientation which continues while the helix is still intact. This feature is expected since α_C_ dynamics are directly implicated in the U1A-RNA binding mechanism. From there on, the overall structure expands. Between 2 and 3 ns, the helix α_A_ exhibits an oscillation between its position in the native state and a considerable displacement, but the antiparallel β-sheet remains assembled. Beyond 3 ns, β_4_ separates and at 4 ns is perpendicular to the β_2_β_3_β_4_ motif. At this point, α_B_ separates more from the remaining core of the structure. At 5 ns, β_4_ has returned and the four-stranded β sheet has reformed but remains expanded relative to the native state. In the next interval of time, β_4_ oscillates between an antiparallel form with β_2_β_3_β_4_ and a form perpendicular to it. These structures represent only the early stages of the unfolding transition state (see below). Overall, the RRM is qualitatively intact but shows a 14% increase in R_g_, i.e., slightly expanded compared to the native state structure. The antiparallel β_1_β_3_β_2_ tertiary motif is still intact after 20 ns. Over the next 10 ns, β_4_ along with α_B_ becomes detached from the β_1_β_3_β_2_ motif and the secondary structure of both dissipates. At 50 ns, a large portion of the structure has unfolded, but interestingly the β_1_β_3_β_2_ remains somewhat intact and α_A_ has returned back into closer proximity to the truncated β-sheet. It is interesting that 2/3 of the RRM motif β_1_α_A_β_2_β_3_α_B_β_4_ are relatively intact in an albeit expanded, molten globule-like form up to 35 ns of MDUF. By 100 ns the tertiary structure has unfolded further, although even at this point vestiges of the partially formed secondary structure remain, and a small but non-native element of tertiary assembly is found. A plot of the time evolution of secondary structure is shown in Figure 9, and clearly shows the persistence of the β_1_α_A_β_2_β_3_ motif. Our results indicate that the folding nucleus of U1A is comprised of a substantial amount of the RRM. The MDUF trajectory for U1A read in the folding direction follows a nucleation-condensation mechanism [67].

**Figure 10:**
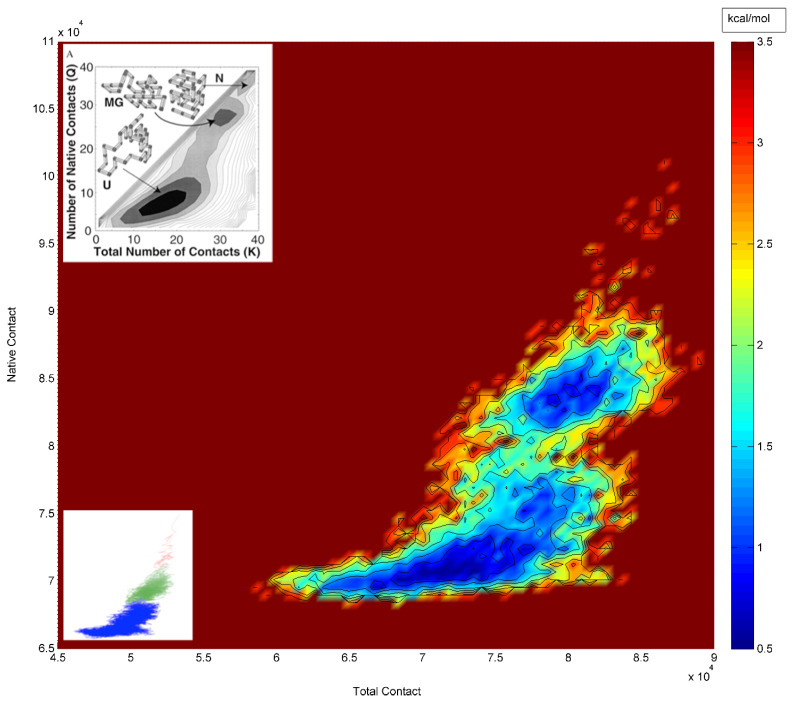
Evolution of secondary structure in the MDUF trajectory #1 of U1A. Key: Yellow: β-sheets. Red: α-helices. This plot was obtained using VMD [68]. Top left insert: Corresponding lattice model results from Pande and Rokhsar [70].

**Figure 11:**
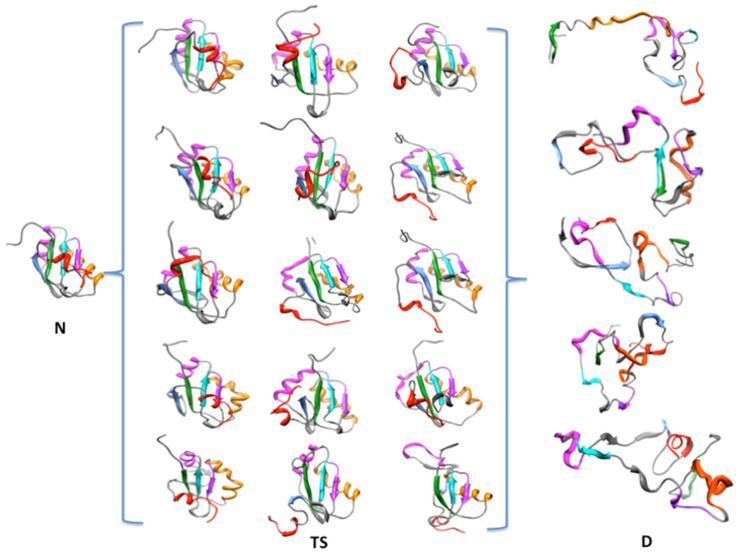
Results from 5 MDUF simulations: the brackets contain an ensemble of transition state structures.

Molten globules are protein structures that retain native-like secondary structures but lack the close packing of the native state ensemble [69]. To explore further if this applies to the transition state ensemble of U1A, we refer to the study of Pande and Rokhsar [65,70], who simulated the folding/unfolding pathway of a lattice model protein using Monte Carlo methods. In their analysis, they plotted the number of contacts in the native state vs. the total number of contacts and found that a molten globule state was well-defined. In Figure 10, we show the corresponding plot from the entire 100 ns MDUF simulation on U1A; Figure 1a of Pande et al. [70] is reproduced in an inset. Their features of the prototype unfolding are found to be well reproduced by our all-atom MDUF simulation on U1A, consistent with the behavior of a molten globular intermediate. The regions indicated to be molten globules by this analysis occur between 1.2 and 35 ns of the 100 ns trajectory, which we take as the demarcation of the upper bound of the extended transition region. Molecular structures from the transition state ensemble including results from all five MDUF simulations are shown in Figure 11. The transitional structures all show by inspection, variations on the theme of protein molten globules. Thus, MDUF simulations predict that the malleable transition state of U1A is an ensemble of molten globule microstates. 

**Figure 12:**
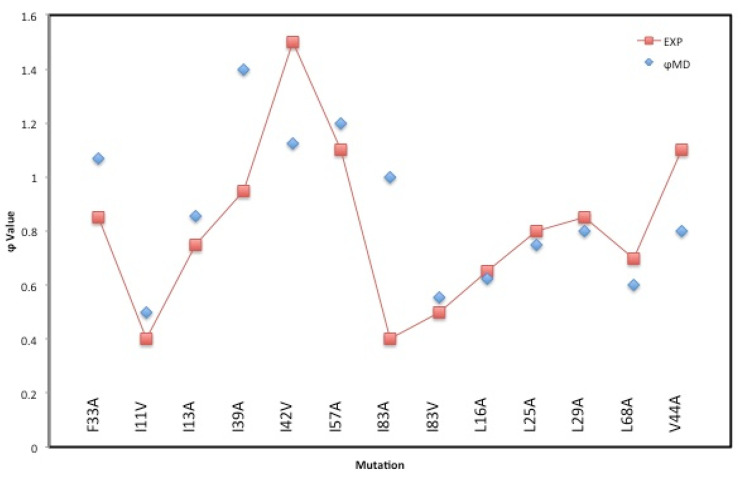
A comparison of experimentally observed [27], and MD calculated φ-values for 13 mutants forms of U1A.

To further validate our MD simulations, we present the results of 100 ns MDUF simulations on the 13 mutant forms of U1A for which the experimentally observed φ-values are measures of how the energetics of protein folding are changed by single-site mutations [27]. Each of these simulations was performed using a protocol analogous to that described for our MD on wild-type U1A. The MD φ-values were calculated from the ratio of the difference between average native contacts in the mutant and wild-type transition states divided by the difference between average native contacts in the folded mutant and wild-type states of U1A [12]. A comparison of the observed and MD calculated results on φ-values is shown in Figure 12. The trends observed are well reproduced by the MD calculated values, especially since the observed results are from chemical denaturation and the calculated results are from thermal denaturation. This supports the idea that the mechanisms of chemical and thermal denaturing processes for small proteins are similar, but this is likely to be system-specific.

## 5. Summary and Conclusions

All-atom, explicit solvent MD thermal unfolding studies on U1A result in a free energy landscape which is consistent with observed kinetics of denaturation in terms of a high energy folding channel. The MD structures corresponding to this region provide a detailed all-atom molecular model for the malleable transition state, with the variation due to sampling different configurations of dynamical elements of the structure, while the RRM core remains intact but slightly expanded. This conforms to the definition of molten globules, and thus our MDUF on U1A indicates the transition state for unfolding is an ensemble of molten globule states. MD calculated results are generally consistent interpretations of experimental data from chevron plots and φ-value analysis. This also provides support for the relevance of free energy landscapes in RMSD space to the protein unfolding problem. The observed progress of the folding is consistent with the expansion of residue-based isodensity surfaces [29] and provides a molecular model in support of the mechanism proposed from φ-values studies that the nucleation motif likely involves a two-sheet helix motif [27,30]. In this project, we compare the results of MD on thermal unfolding with experimental results on chemical denaturation, and the general agreement obtained, supports the idea that the folding pathway is similar in the two cases [5,39,71,72]. To further investigate this point, a corresponding MD study of the chemical denaturation of U1A and a corresponding comparison with experimental data is currently in progress. A more detailed analysis of the kinetics is being carried out using the program Wordom [73,74].

*Note added in proof*: A referee has asked that we comment on the sensitivity of our results to our choice of the AMBER force field. Rueda, Ferrer-Costa, Meyer and Orozco [75] have reported an extensive longitudinal comparison of results from the four most popular MD force fields including AMBER, which indicates that MD results at the level of resolution of our study are expected to be force field independent.
